# Perioperative immunotherapy in resectable gastric adenocarcinoma: a critical review of current evidence

**DOI:** 10.1590/0102-672020260000053e1982

**Published:** 2026-11-09

**Authors:** Valentina Patino-Perez, Sarita Castrillón-Arango, Yensy Paola Botello-Valencia, Edwin Oveimar Muñoz-Ruiz, Rosa Amalia Dueñas-Cuellar

**Affiliations:** 1 Universidad del Cauca, Facultad de Ciencias de la Salud, Especialización en Cirugía General, Departamento de Ciencias Quirúrgicas – Popayán (Cauca), Colombia.; 2 Universidad del Cauca, Facultad de Ciencias de la Salud, Departamento de Patología, Grupo de Investigación en Inmunología y Enfermedades Infecciosas – Popayán (Cauca), Colombia.

**Keywords:** Stomach neoplasms, Immunotherapy, Perioperative period, Immune checkpoint inhibitors, Gastrectomy, Neoplasias gástricas, Imunoterapia, Período perioperatório, Inibidores de checkpoint imunológico, Gastrectomia

## Abstract

**Background:**

Gastric cancer ranks fifth in global cancer incidence and fourth in mortality. In patients with gastric adenocarcinoma, perioperative chemotherapy with fluorouracil, leucovorin, oxaliplatin, and docetaxel, the FLOT regimen, constitutes the current therapeutic standard. Incorporating immune checkpoint inhibitors into perioperative schemes has emerged as a promising strategy, although its clinical and surgical impact requires critical appraisal in light of current evidence.

**Aims:**

To critically analyze contemporary scientific evidence on incorporating perioperative immunotherapy into resectable gastric adenocarcinoma management, evaluating its impact on oncologic outcomes, perioperative safety, and its role within current therapeutic algorithms.

**Methods:**

A critical narrative review through systematic searches in PubMed/MEDLINE, Embase, and Cochrane Library using relevant terms of the Medical Subject Headings (MeSH) thesaurus.

**Results:**

The MATTERHORN trial demonstrated that the addition of durvalumab to FLOT significantly improves event-free survival (hazard ratio 0.71; 95%CI 0.58–0.86; p<0.001) and overall survival (hazard ratio 0.78; 95%CI 0.63–0.96; p=0.021), with a pathological complete response rate of 19.2% versus 7.2%, without increased surgical morbidity or mortality. The KEYNOTE-585 trial demonstrated improved pathological complete response with pembrolizumab, without significant overall survival benefit. Microsatellite instability-high, human epidermal growth factor receptor 2, and programmed death ligand 1 biomarkers are key determinants in therapeutic selection. Perioperative immune-related adverse events require specific surveillance.

**Conclusions:**

Adding durvalumab to FLOT establishes a new perioperative standard with level I evidence. Preoperative molecular characterization is key to decision-making. Integrating immunotherapy does not compromise the feasibility of gastrectomy with extended second-level lymphadenectomy, but requires specific expertise to manage immune-related toxicities.

## ARTICLE HIGHLIGHTS

- Durvalumab added to the fluorouracil, leucovorin, oxaliplatin, and docetaxel (FLOT) regimen significantly improves event-free survival and overall survival, with pathological complete response, without increased surgical morbidity.
- The KEYNOTE-585 trial showed improved pathological complete response with pembrolizumab but failed to demonstrate overall survival benefit, confirming that immunotherapy efficacy is not uniform across agents or unselected populations.
- Preoperative molecular profiling of MSI-H, PD-L1, and HER2 status is now the central axis for individualized therapeutic decision-making in resectable gastric adenocarcinoma.
- Perioperative immunotherapy does not compromise the feasibility of gastrectomy with extended second-level lymphadenectomy but requires specific institutional expertise to manage immune-related adverse events.

## Summary

**CENTRAL MESSAGE:**

Gastric cancer continues to represent a significant oncologic burden worldwide, ranking fifth in incidence and fourth in mortality. Its high lethality is related to the frequent diagnosis at locally advanced stages and its aggressive biological behavior, particularly in high-risk regions such as East Asia and Latin America. In this context, radical gastrectomy with extended second-level lymphadenectomy constitutes the cornerstone of curative-intent treatment, providing adequate locoregional control and precise staging. Incorporating multimodal strategies, such as perioperative chemotherapy, has demonstrated improved oncologic outcomes compared with surgery alone. The associated immunotherapy based on immune checkpoint inhibitors has emerged as a promising strategy.

**PERSPECTIVES:**

The approval of durvalumab in combination with the fluorouracil, leucovorin, oxaliplatin, and docetaxel (FLOT) regimen establishes a new perioperative standard with level I evidence, while the results of KEYNOTE-585 underscore that this benefit is neither uniform across agents nor applicable to unselected populations. Preoperative molecular characterization — particularly the determination of MSI-H, PD-L1, and HER2 — ceases to be a staging exercise to become the axis upon which the individual therapeutic strategy is constructed: it defines the systemic regimen, anticipates the probability of pathological complete response, and, in the MSI-H subgroup, opens the possibility of exploring - within the strict framework of research protocols - whether gastrectomy is necessary when treatment response is complete.

## VISUAL ABSTRACT

**Figure:**
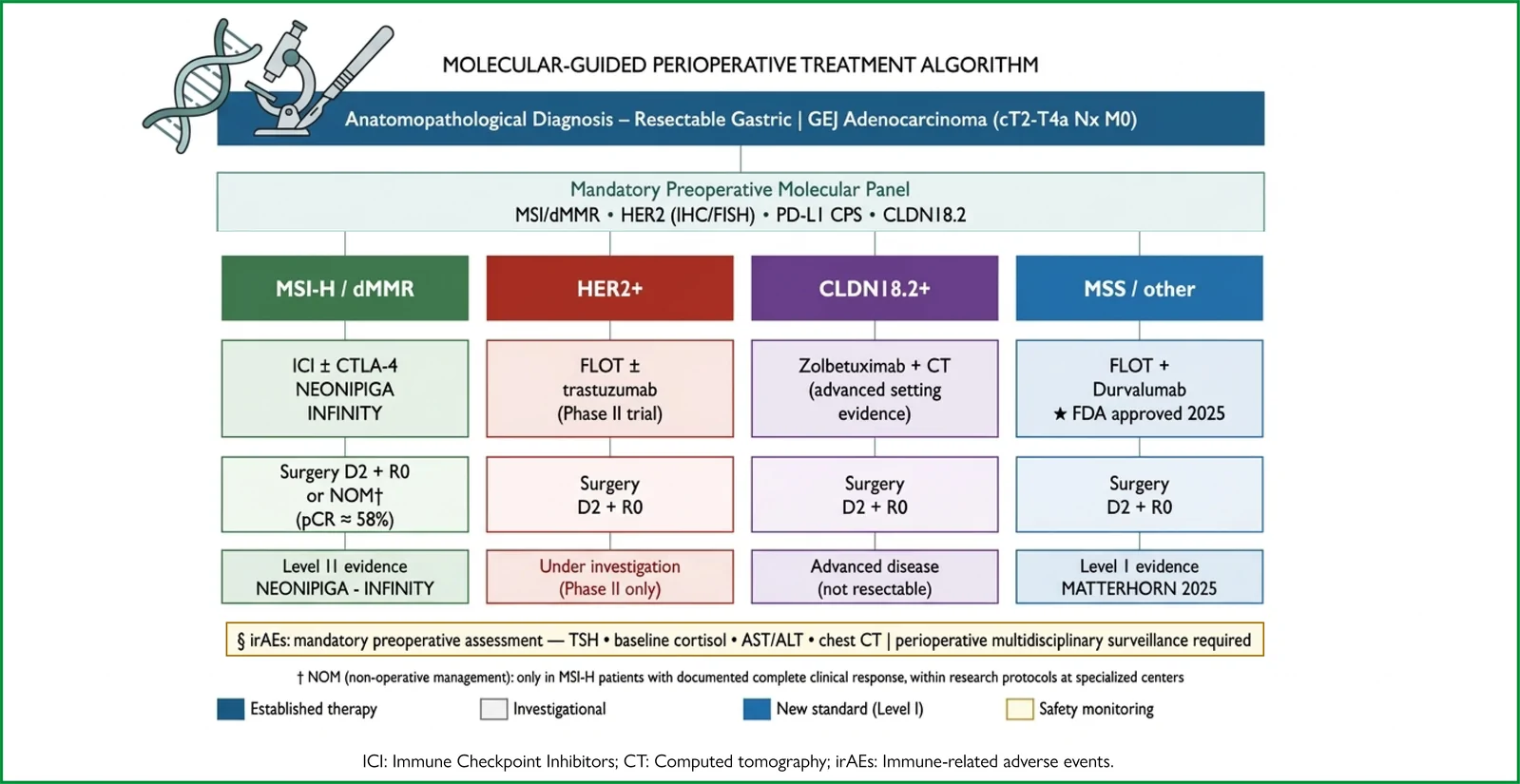

## RESUMO

**Racional:**

O câncer gástrico representa a quinta neoplasia em incidência mundial e a quarta em mortalidade. Em pacientes com adenocarcinoma gástrico, a quimioterapia perioperatória com o esquema FLOT constitui o padrão terapêutico vigente. A incorporação de inibidores de pontos de controle imunológico ao esquema perioperatório emergiu como estratégia promissora, embora seu impacto clínico e cirúrgico exija análise crítica à luz da evidência atual.

**ObjetivoS:**

Analisar criticamente a evidência científica contemporânea sobre a integração da imunoterapia perioperatória no manejo do adenocarcinoma gástrico ressecávei, avaliando seu impacto nos resultados oncológicos, segurança perioperatória e papel nos algoritmos terapêuticos atuais.

**Métodos:**

Revisão narrativa crítica mediante busca nas bases PubMed/MEDLINE, Embase e Cochrane Library, utilizando termos MeSH relevantes.

**Resultados:**

O ensaio MATTERHORN demonstrou que a adição de durvalumabe ao FLOT melhora significativamente a sobrevida livre de eventos (HR 0,71; IC95% 0,58–0,86; p<0,001) e a sobrevida global (HR 0,78; IC95% 0,63–0,96; p=0,021), com taxa de resposta patológica completa de 19,2% versus 7,2%, sem aumento da morbimortalidade cirúrgica. O KEYNOTE-585 demonstrou aumento da resposta patológica completa com pembrolizumabe, sem benefício significativo na sobrevida global. Os biomarcadores MSI-H, HER2 e PD-L1 são determinantes na seleção terapêutica. Os eventos adversos imunológicos perioperatórios requerem vigilância específica.

**Conclusões:**

A combinação durvalumabe mais FLOT estabelece um novo padrão perioperatório com nível I de evidência. A caracterização molecular pré-operatória é o eixo central da tomada de decisão. A integração da imunoterapia não compromete a viabilidade da gastrectomia com linfadenectomia D2, mas exige competências no manejo das toxicidades imunológicas.

## INTRODUCTION

Gastric cancer continues to represent a significant oncologic burden worldwide, ranking fifth in incidence and fourth in mortality^9^. Its high lethality is related to the frequent diagnosis at locally advanced stages and its aggressive biological behavior, particularly in high-risk regions such as East Asia and Latin America. In this context, radical gastrectomy with extended second-level (D2) lymphadenectomy constitutes the cornerstone of curative-intent treatment, providing adequate locoregional control and precise staging. However, surgery as a single modality proves insufficient in locally advanced disease, where systemic recurrence remains the leading cause of mortality, reflecting the early disseminated nature of this neoplasm^7,21,49^.

Multimodal strategies substantially transformed resectable gastric cancer management. Perioperative chemotherapy has demonstrated improved oncologic outcomes compared with surgery alone, establishing a paradigm shift in treatment^15^. Subsequently, optimization of chemotherapy regimens, and particularly the introduction of the FLOT (fluorouracil, leucovorin, oxaliplatin, and docetaxel) regimen, consolidated this approach as the contemporary standard, improving survival and complete resection rates^2^. However, despite these advances, a significant proportion of patients continues to develop tumor recurrence, highlighting the limitations of conventional chemotherapy and the need for more targeted therapeutic strategies^29,36^.

In parallel, advances in molecular characterization have enabled the identification of tumor subtypes with relevant prognostic and predictive implications. Human epidermal growth factor receptor 2 (HER2) amplification has demonstrated clinical value for selecting targeted therapies^5^, while microsatellite instability-high (MSI-H) is associated with better prognosis and a differential response to chemotherapy, supporting the development of more personalized therapeutic approaches^42,56^. These findings have redefined decision-making, in which tumor biology plays a central role.

In this context, immunotherapy based on immune checkpoint inhibitors has emerged as a promising strategy. Its efficacy in advanced disease has driven its evaluation in the perioperative setting to improve tumor eradication and control of micrometastatic disease^29,60^. Recent trials have explored its integration into perioperative chemotherapy, demonstrating relevant clinical activity; however, its definitive impact on overall survival and its role within therapeutic algorithms remain under evolution^24,53^.

Therefore, the objective of this review is to critically analyze the contemporary scientific evidence on integrating perioperative immunotherapy into resectable gastric and gastroesophageal junction (GEJ) cancer management, evaluating its impact on oncologic outcomes, its safety from a surgical perspective, and its role within current therapeutic algorithms to provide surgeons with an updated conceptual framework to optimize multidisciplinary decision-making and improve survival in patients eligible for curative treatment.

## METHODS

A critical narrative review was conducted through systematic searches in PubMed/MEDLINE, Embase, and Cochrane Library (January 2001–April 2026), combining Medical Subject Headings (MeSH) terms with Boolean operators. The main terms included: gastric cancer, perioperative immunotherapy, immune checkpoint inhibitors, neoadjuvant treatment, and resectable disease. The search was supplemented by manual reference review, consultation of the current European Society of Medical Oncology (ESMO) and National Comprehensive Cancer Network (NCCN) guidelines, and consultation of ClinicalTrials.gov and ICTRP registries.

Phase II-III clinical trials, meta-analyses, systematic reviews, and international guidelines on immune checkpoint inhibitors (anti-programmed cell death protein 1 (anti-PD-1); anti-programmed death-ligand 1 (anti-PD-L1); and anti-cytotoxic T-lymphocyte-associated protein 4 (anti-CTLA-4)) in resectable gastric or GEJ adenocarcinoma were included. Phase I studies without efficacy data, studies limited to metastatic disease, case series with fewer than ten patients, and congress communications without subsequent indexed publication were excluded.

## RESULTS

All authors independently selected the studies. Discrepancies were resolved by consensus. Data extraction included study design, population, intervention, comparator, and primary outcomes (overall survival, event-free survival, pathological complete response, R0 resection, perioperative morbidity and mortality, and grade 3 adverse events).

Given the heterogeneity across studies, the authors narratively synthesized the results, reporting the hazard ratio (HR), the 95% confidence intervals (CI), and p-values as published in the original studies.

## DISCUSSION

### Epidemiology and clinical relevance of resectable gastric cancer

Gastric cancer ranks fifth in incidence and fourth in global cancer mortality, with approximately 968,000 new cases and 660,000 deaths estimated in 2022, concentrated primarily in East Asia, Eastern Europe, and Latin America^9^. Although the incidence of distal tumors has progressively declined, a relative increase in proximal adenocarcinomas and GEJ tumors has been observed, associated with obesity and gastroesophageal reflux disease^7,9,21^. Clinically, the majority of patients in Western countries are diagnosed at locally advanced stages (cT2-T4 and/or regional lymph node involvement), in contrast to Japan and South Korea, where national endoscopic screening programs have substantially improved early detection and survival^5,14,21,22^.

### Surgical principles

Radical gastrectomy with D2 lymphadenectomy constitutes the cornerstone of curative treatment for resectable gastric cancer, ideally performed in high-volume centers within multidisciplinary teams^3,33^. The central oncologic objective is R0 resection — absence of microscopic residual disease — the most important prognostic determinant after curative surgery^13^. The extent of gastric resection is individualized according to tumor location: subtotal gastrectomy for distal tumors and total gastrectomy for proximal or diffuse tumors, provided adequate negative margins are achieved^13,33,58^.

D2 lymphadenectomy is the standard of care in locally advanced disease, recommended by ESMO, NCCN, and current Japanese guidelines^2,33,27^. Randomized trials and the 15-year follow-up of the Italian Gastric Cancer Study Group confirm superior disease-specific survival with D2 over more limited dissections^17,35,48^. Evaluation of 16 lymph nodes is recommended to ensure adequate pathological staging^13^. In early-stage disease with low risk of lymph node involvement, endoscopic submucosal dissection (ESD) and endoscopic mucosal resection (EMR) offer outcomes comparable to radical surgery with lower morbidity and mortality^18,23,27,33^.

### Evolution toward multimodal treatment

The treatment of resectable gastric cancer progressively evolved from surgery alone toward a multimodal model, driven by recurrence rates of 40–70% after apparently complete resections, attributed to subclinical micrometastatic disease^16^. The INT-0116 trial (2001) established adjuvant chemoradiotherapy as the Western standard, demonstrating significant improvement in overall survival (median overall survival (OS) 36 vs. 27 months; HR 1.35; p=0.005)^34,55^. In Asia, the ACTS-GC study consolidated adjuvant S-1 after D2 gastrectomy (5-year OS 71.7% vs. 61.1%; HR 0.669; p=0.0005)^46,47^, and the CLASSIC trial confirmed the benefit of adjuvant oxaliplatin and capecitabine (CAPOX) (5-year event-free survival 68 vs. 53%; HR 0.58; p<0.001)^4^.

The concept of perioperative chemotherapy was established by the MAGIC trial (2006), which demonstrated improved 5-year overall survival with perioperative epirubicin, cisplatin, and fluorouracil (ECF) versus surgery alone (36 vs. 23%; HR 0.75; p=0.009), with higher R0 resection rates^15^. The FNCLCC/FFCD trial confirmed these results with perioperative cisplatin and fluorouracil (5-year OS 38 vs. 24%; HR 0.69; p=0.02)^59^. The definitive advance came with the FLOT4-AIO trial, demonstrating superiority of the FLOT regimen over ECF/epirubicin, cisplatin, and capecitabine (ECX) (median OS 50 vs. 35 months; HR 0.77; p=0.012; R0 85 vs. 78%; pathological complete response 16 vs. 6%), consolidating FLOT as the current perioperative standard^2^.

Persistent systemic recurrence drove the evaluation of immune checkpoint inhibitors in earlier disease settings. The KEYNOTE-059 and CheckMate-649 studies demonstrated the benefit of anti-PD-1 agents in advanced disease with programmed death ligand 1 (PD-L1) expression, establishing the biological rationale for their perioperative evaluation^11,20,25^. Recent trials such as MATTERHORN and KEYNOTE-585 explored their integration with perioperative chemotherapy, with heterogeneous results that underscore the importance of molecular patient selection^24,26,51^.

### Biological basis and tumor microenvironment

Gastric cancer develops immune evasion mechanisms centered on the programmed cell death protein 1 (PD-1)/PD-L1 signaling pathway. The PD-1 receptor, expressed on activated T lymphocytes, interacts with PD-L1 on tumor cells and tumor-infiltrating immune cells, inhibiting cytotoxic T lymphocyte activation and promoting functional exhaustion of the antitumor immune response^29,60^. PD-L1 expression in the gastric tumor microenvironment represents an adaptive mechanism of immune resistance induced by interferon-γ, establishing a negative feedback circuit that facilitates tumor progression^1,8,10,19,28,38–40^.

Clinically, PD-L1 expression is assessed by immunohistochemistry using the Combined Positive Score (CPS), which quantifies PD-L1 expression in both tumor cells and tumor-infiltrating immune cells relative to the total number of viable tumor cells^19^. In the KEYNOTE-059 study, PD-L1-positive tumors showed higher objective response rates to pembrolizumab than PD-L1-negative tumors (15.5% vs. 6.4%), suggesting that PD-L1 expression reflects an immunologically active tumor microenvironment susceptible to PD-1/PD-L1 pathway blockade^11,40^.

Blockade of the PD-1/PD-L1 interaction through monoclonal antibodies restores cytotoxic T lymphocyte activation and reactivates antitumor immune surveillance^1^. In the perioperative setting, an intact primary tumor acts as a continuous source of tumor antigens, potentiating systemic immune activation and facilitating elimination of subclinical micrometastatic disease. PD-L1 expression assessed by CPS allows identification of patient subgroups with greater likelihood of benefit, optimizing therapeutic selection within contemporary treatment algorithms^24,53,57^.

### Biomarkers as the cornerstone of precision medicine

Molecular characterization of gastric cancer has enabled the identification of biomarkers with prognostic and predictive implications, facilitating the transition toward a personalized therapeutic approach (Table 1). Among the biomarkers with the greatest clinical impact are MSI-H, HER2 amplification, and claudin 18.2 (CLDN18.2) expression, in addition to the PD-1/PD-L1 pathway already discussed^5,10,52^.

**Table 1:** Clinically relevant biomarkers in gastric cancer.

Biomarker	Alteration	Approximate frequency	Detection method	Prognostic implication	Predictive implication	Associated therapies
MSI-H/dMMR	Microsatellite instability due to DNA mismatch repair deficiency	5–10%	IHC (MLH1, MSH2, MSH6, PMS2), PCR or NGS	Better prognosis in localized disease	High response to immunotherapy	PD-1 inhibitors (pembrolizumab, nivolumab). ORR ~40–60% in advanced disease
HER2	HER2 tyrosine kinase receptor overexpression/amplification	10–20% (more frequent in intestinal type)	IHC + confirmatory FISH/ISH	Associated with more aggressive disease (variable)	Predicts response to anti-HER2 therapy	Trastuzumab + CT (ToGA: OS 13.8 vs 11.1 months; HR 0.74; p=0.0046)
PD-L1 (CPS)	PD-L1 expression in tumor/immune cells (Combined Positive Score)	Variable (CPS ≥1 in ~60%)	IHC (22C3, 28-8)	Not clearly prognostic	Predicts benefit from immunotherapy	Nivolumab + CT (CheckMate-649: HR 0.71 in CPS ≥5); Pembrolizumab in high CPS
CLDN18.2	Claudin 18.2 overexpression (tight junction protein)	30–40%	IHC	Under investigation (possible specific phenotype)	Predicts response to anti-CLDN18.2 targeted therapies	Zolbetuximab + CT (SPOTLIGHT: HR 0.75 OS; p<0.01)

MSI-H: microsatellite instability-high; dMMR: DNA mismatch repair; HER2: human epidermal growth factor receptor 2; PD-L1: programmed death ligand 1; CPS: Combined Positive Score; CLDN18.2: claudin 18.2; IHC: immunohistochemistry; PCR: polymerase chain reaction; NGS: next-generation sequencing; FISH: fluorescence in situ hybridization; ISH: in situ hybridization; ORR: objective response rate; OS: overall survival; HR: hazard ratio; CT: chemotherapy.

MSI-H represents the most relevant molecular biomarker in gastric cancer. Deficiency in deoxyribonucleic acid (DNA) mismatch repair mechanisms (dMMR) generates accumulation of somatic mutations and tumor neoantigens that induce a robust immune response, with intense lymphocytic infiltration and high probability of response to immune checkpoint inhibitors^10,11,31,42^. An individual patient data meta-analysis demonstrated 5-year overall survival rates >80% in MSI-H patients treated with surgery, with a possible unfavorable effect of conventional perioperative chemotherapy, justifying the development of immunotherapy-based alternative therapeutic strategies for this subgroup^12,30,31,42^.

HER2 amplification, present in 15-20% of gastric and GEJ adenocarcinomas, was the first validated therapeutic target in this disease. The ToGA trial demonstrated that adding trastuzumab to chemotherapy significantly improves overall survival (median OS 13.8 vs. 11.1 months; HR 0.74; 95%CI 0.60–0.91; p=0.0046) in HER2-positive patients, with its routine determination incorporated into diagnostic algorithms^5,6,10,33,44^.

More recently, CLDN18.2 has emerged as a novel promising therapeutic target in gastric cancer. This protein is part of tight junctions, and, under normal conditions, its expression is limited to differentiated gastric epithelium. During malignant transformation, alteration of the epithelial architecture exposes this epitope on the surface of tumor cells, making it accessible to targeted therapies^37,45,54^. The phase III SPOTLIGHT trial demonstrated that the addition of zolbetuximab to oxaliplatin- and fluoropyrimidine-based chemotherapy in CLDN18.2-positive advanced gastric cancer patients was associated with significant improvements in both progression-free and overall survival^52^. Similar results were subsequently reported in the phase III GLOW trial, consolidating CLDN18.2 as one of the new pillars of molecular stratification in gastric cancer^50^.

### Current clinical evidence

The development of immunotherapy in the perioperative treatment of resectable gastric cancer represents one of the most relevant advances within the contemporary multimodal model (Figure 1). However, its clinical and surgical impact must be interpreted in light of the totality of available evidence and in strict relation to current clinical practice recommendations. Unlike the FLOT4-AIO trial, which established an unequivocal overall survival benefit and consolidated the FLOT regimen as the perioperative reference standard, studies with immune checkpoint inhibitors have shown heterogeneous results and, in some cases, results dependent on the molecular profile of the tumor, which conditions their applicability in current clinical practice^2^.

**Figure 1:**
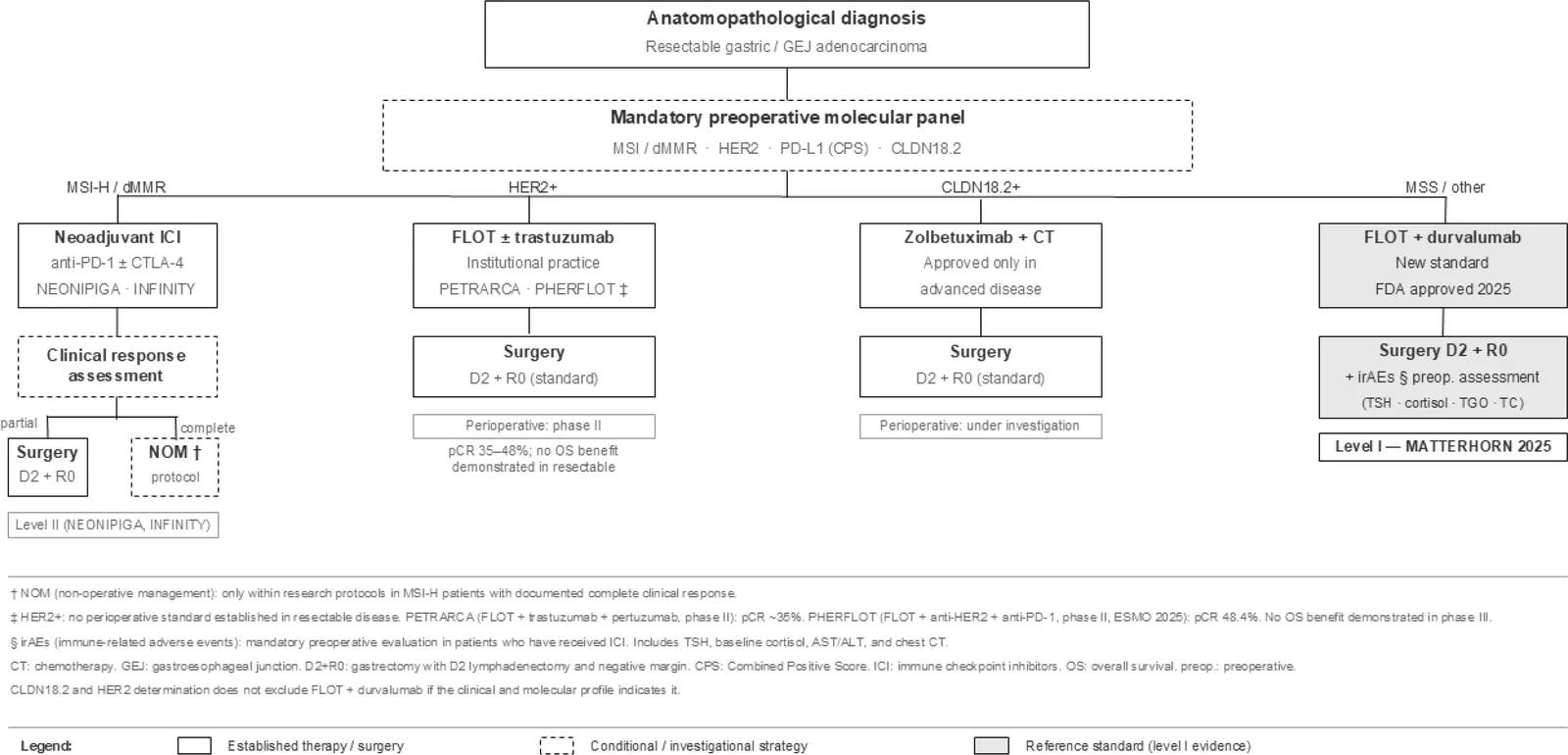
Perioperative therapeutic algorithm for resectable gastric and gastroesophageal junction adenocarcinoma, stratified by molecular profile. The mandatory preoperative molecular panel (MSI-H/dMMR, HER2, PD-L1, and CLDN18.2) is detailed as the central axis for multidisciplinary decision-making. MSI-H/dMMR subgroup: Exclusive or dual neoadjuvant immunotherapy (immune checkpoint inhibitors) strategies (anti-PD-1/anti-CTLA-4) evaluated in research settings (NEONIPIGA and INFINITY trials). Following clinical response assessment, patients are allocated to radical surgical resection or selected non-operative management (NOM) protocols with organ preservation. HER2+ subgroup: Integration of the FLOT regimen combined with targeted therapy (trastuzumab) based on institutional practice and phase II trials (PETRARCA, PHERFLOT), followed by radical surgery. CLDN18.2+ subgroup: Use of zolbetuximab in combination with chemotherapy, currently approved only for advanced/metastatic disease (SPOTLIGHT and GLOW trials) and under investigation for the perioperative setting. MSS/Variable expression/Other subgroup: New perioperative treatment standard (Level I evidence, MATTERHORN trial 2025) — FDA-approved FLOT plus durvalumab, followed by radical gastrectomy with D2 lymphadenectomy and R0 margin achievement.

Abbreviations: FLOT: Fluorouracil, Leucovorin, Oxaliplatin and Docetaxel; ICI: Immune Checkpoint Inhibitors; MSI-H/dMMR: High Microsatellite Instability/Deficient Mismatch Repair; HER2: Human Epidermal Growth Factor Receptor 2; PD-L1 (CPS): Programmed Death-Ligand 1 (Combined Positive Score); CLDN18.2: Claudin 18.2; NOM: Non-Operative Management; D2: Extended second-level lymphadenectomy; R0: Complete resection without microscopic residual disease; FDA: Food and Drug Administration of the United States.

In current clinical practice, the international ESMO and NCCN guidelines continue to recommend FLOT-based perioperative chemotherapy as the standard of care for patients with resectable gastric or GEJ adenocarcinoma in good functional status^33^. This approach has demonstrated significant improvement in overall survival compared to prior epirubicin-based regimens (median OS 50 vs. 35 months; HR 0.77; 95%CI 0.63–0.94; p=0.012), as well as higher R0 resection rates (85% FLOT vs. 78% ECF/ECX in intention-to-treat (ITT) population) and pathological complete response, constituting the therapeutic cornerstone against which new strategies are evaluated^2^. From a surgical perspective, this regimen has optimized resectability and locoregional control without compromising operative safety, establishing a solid reference point for the incorporation of additional therapies.

In the metastatic setting, the CheckMate-649 trial demonstrated that the addition of nivolumab to fluoropyrimidine- and platinum-based chemotherapy significantly improves overall survival in patients with HER2-negative gastric, GEJ, or esophageal adenocarcinoma with PD-L1 CPS=5 (median OS 14.4 versus 11.1 months; HR 0.71; 95%CI 0.61–0.83; p<0.0001), leading to its adoption as the first-line standard in advanced disease^25^. However, extrapolation of these results to the perioperative setting requires caution: CheckMate-649 exclusively included HER2-negative patients with CPS=5, while in the resectable setting, biomarker-based selection remains heterogeneous across available trials and enrichment strategies are not yet fully defined.

In the resectable setting, the phase III KEYNOTE-585 trial evaluated the addition of pembrolizumab to perioperative chemotherapy in patients with locally advanced gastric or GEJ adenocarcinoma. The final analysis, with a median follow-up of 59.9 months, showed that the addition of pembrolizumab was associated with an increase in the pathological complete response rate (12.9 vs. 2.0%; p<0.00001), but without demonstrating statistically significant benefit in event-free survival — the primary endpoint — nor in overall survival (median OS 71.8 versus 55.7 months; HR 0.86; 95%CI 0.71–1.06)^53^. This finding limited the adoption of pembrolizumab in this context: current ESMO and NCCN guidelines do not recommend perioperative immunotherapy routinely outside of clinical trials^3,33^. From a surgical perspective, although the increase in pathological complete response suggests greater biological activity of the combined regimen, the absence of consistent correlation with survival outcomes limits its impact on operative decision-making in unselected populations.

In contrast, the phase III MATTERHORN trial, published in The New England Journal of Medicine in 2025, has provided the most relevant and definitive evidence in this setting^24^. This study evaluated the addition of the PD-L1 inhibitor durvalumab to the FLOT regimen in 948 patients with resectable gastric or GEJ adenocarcinoma. The primary endpoint analysis, with a median follow-up of 31.5 months, demonstrated a statistically significant improvement in event-free survival (HR 0.71; 95%CI 0.58–0.86; p<0.001), with a 2-year event-free survival rate of 67.4% in the durvalumab arm versus 58.5% in the chemotherapy-alone arm. The pathological complete response rate increased from 7.2% with FLOT plus placebo to 19.2% with FLOT plus durvalumab. The final overall survival analysis, presented at the 2025 ESMO congress, confirmed a significant benefit (HR 0.78; 95%CI 0.63–0.96; p=0.021). Based on these results, the United States Food and Drug Administration approved durvalumab in combination with FLOT as perioperative treatment for resectable gastric and GEJ adenocarcinoma in 2025. From a surgical perspective, MATTERHORN also demonstrated that the addition of durvalumab did not increase perioperative morbidity or mortality and did not generate clinically significant delays in surgery, with comparable surgical delay rates between both arms (~10%).

A central element in the integration of perioperative immunotherapy is adequate patient selection through molecular biomarkers. Clinical guidelines recommend MSI and PD-L1 assessment in advanced disease, but their role in the perioperative setting has not yet been defined. Evidence suggests that MSI-H tumors may benefit substantially from immunotherapy: an individual patient data meta-analysis demonstrated 5-year overall survival rates >80% in MSI-H gastric cancer patients treated with surgery, with a possible limited or adverse benefit from conventional chemotherapy, justifying the development of immunotherapy-based alternative strategies for this subgroup^42^. It is noteworthy that none of the pivotal perioperative trials were designed with strict MSI or CPS enrichment criteria, which may partly explain the heterogeneity of results observed in unselected populations. This context reinforces the need to incorporate biological and molecular variables in therapeutic planning, modifying the traditional paradigm of surgical decisions based exclusively on anatomical and clinical criteria.

Finally, as for perioperative safety, data from both the MATTERHORN and the KEYNOTE-585 trials indicate that incorporating immunotherapy does not compromise the technical feasibility of gastrectomy with D2 lymphadenectomy or significantly increase postoperative morbidity and mortality. This finding is consistent with the accumulated experience in other solid tumors treated with neoadjuvant immunotherapy. From a surgical perspective, R0 resection continues to be a priority with adequate lymphadenectomy. Perioperative immunotherapy, when indicated, can be incorporated into protocols without modifying the fundamental principles of oncologic surgery.

### Surgical implications

Evidence from the phase III MATTERHORN and KEYNOTE-585 clinical trials consistently demonstrates that the incorporation of immune checkpoint inhibitors into perioperative treatment does not increase conventional surgical risk or compromise the technical feasibility of gastrectomy with D2 lymphadenectomy. In the MATTERHORN trial, the completed surgical resection rate was 92% in both treatment groups — FLOT plus durvalumab and FLOT plus placebo — with no clinically relevant differences in serious postoperative complications or perioperative mortality^24^. Similarly, the final analysis of KEYNOTE-585 confirmed comparable surgical resection and grade 3–5 postoperative complication rates between pembrolizumab and placebo groups, with more than 90% of patients in both arms completing the planned resection^53^. These data are reassuring, given that immune checkpoint inhibitor-induced immune activation does not generate inflammatory or fibrotic phenomena that impair surgical dissection or increase anastomotic risk. The results are consistent with the accumulated experience in other solid tumors approached with neoadjuvant immunotherapy — including lung, esophageal, and breast cancer — where no clinically relevant increase in conventional surgical complications has been observed^61^. A recent meta-analysis confirmed that the addition of immunotherapy does not increase the overall risk of grade 3–5 treatment-related adverse events, postoperative complication rates, or the proportion of patients who do not achieve resection^32^.

Regarding surgical planning, both trials demonstrated that immunotherapy does not generate clinically significant delays in surgery. In MATTERHORN, surgical delay rates were approximately 10% in both arms, with no differences between groups^24^. This confirms that the incorporation of durvalumab or pembrolizumab into the perioperative regimen does not interfere with surgical scheduling or the optimal therapeutic window for resection. Available evidence therefore supports that perioperative immunotherapy, when indicated, can be safely integrated into multidisciplinary protocols without modifying the fundamental principles of oncologic resection: R0 margin and adequate D2 lymphadenectomy as central prognostic determinants.

While conventional surgical morbidity is not increased with immunotherapy, surgeons working with these patients must be aware of an emerging and specific risk of this therapeutic era: perioperative immune-related adverse events (irAEs). Unlike classic surgical complications such as hemorrhage, anastomotic leak, and surgical site infection, irAEs derive from systemic immune activation induced by immune checkpoint inhibitors (and can affect virtually any organ, with predilection for the endocrine, pulmonary, gastrointestinal, and hepatic systems^41^.

Quantitatively, the previously mentioned meta-analysis demonstrated that the addition of immune checkpoint inhibitors to perioperative chemotherapy is associated with a significantly higher risk of grade 3 irAEs compared with chemotherapy alone (risk ratio (RR) 2.88; 95%CI 1.95–4.24)^32^. This increase in serious irAEs does not translate into higher perioperative mortality, as demonstrated by MATTERHORN and KEYNOTE-585 data, but does require specific surveillance and management protocols that the surgical team must be familiar with.

Clinically, irAEs represent a diagnostic challenge in the perioperative period due to their overlap with conventional surgical complications. Postoperative fatigue and hypotension may correspond to hemorrhagic or infectious complications as well as to adrenal insufficiency from an endocrine irAE; postoperative fever may be infectious or reflect immune pneumonitis; transaminase elevation may be of surgical hepatic origin or immune-related hepatitis. Recent publications from teams specializing in gastric surgery with neoadjuvant immunotherapy have described cases of irAEs — including pneumonitis, adrenal insufficiency, and colitis — that manifested or recurred in the immediate postoperative period, generating delays in diagnosis and management^41^.

In response to this emerging risk, clinical practice at reference centers has begun incorporating specific preoperative evaluation protocols for patients who have received neoadjuvant immunotherapy. These protocols include, in addition to routine hematological assessment: thyroid function and baseline cortisol, detailed liver function tests, baseline chest computed tomography to detect subclinical pneumonitis, and cardiac evaluation when clinically indicated^41^. In the postoperative period, surveillance must extend beyond the conventional window of surgical complications, with a high index of suspicion for irAEs in the presence of atypical symptoms or clinical evolution inconsistent with the expected surgical course. The management of these events, based on corticosteroid immunosuppression and, in severe cases, additional immunomodulators, requires coordinated participation of the surgical team with medical oncology and specialized internists.

The magnitude of complete pathological responses observed with immunotherapy in MSI-H molecular subgroups raises a conceptually relevant question: can complete tumor eradication induced by systemic treatment support, in selected cases, the omission of radical gastrectomy? This question, already explored in MSI-H rectal cancer, has driven prospective trials specifically in gastric cancer with this molecular profile.

The GERCOR NEONIPIGA trial demonstrated exceptional pathological complete response rates (58.69%; 90%CI 41.8–74.1%) with neoadjuvant nivolumab plus ipilimumab in MSI-H/dMMR patients, with some cases declining surgery and remaining recurrence-free during initial follow-up^3^. The INFINITY study prospectively explored the omission of gastrectomy in complete responders after tremelimumab and durvalumab, reporting gastrectomy-free survival of 64.2% in the non-operative management cohort^43^. However, follow-up remains limited, and questions persist regarding response durability; non-operative management must currently be considered exclusively within research protocols at specialized centers, as gastrectomy with D2 lymphadenectomy continues to be the indispensable curative standard for most patients.

## CONCLUSIONS

Accumulated evidence marks an inflection point in the management of resectable gastric adenocarcinoma. The approval of durvalumab in combination with FLOT establishes a new perioperative standard with level I evidence, while the results of KEYNOTE-585 underscore that this benefit is neither uniform across agents nor applicable to unselected populations. Preoperative molecular characterization — particularly the determination of MSI-H, PD-L1, and HER2 — ceases to be a staging exercise to become the axis upon which the individual therapeutic strategy is constructed: it defines the systemic regimen, anticipates the probability of pathological complete response, and, in the MSI-H subgroup, opens the possibility of exploring — within the strict framework of research protocols — whether gastrectomy is always necessary when treatment response is complete.

From a surgical perspective, this paradigm shift does not displace surgery from the center of curative treatment, but adds concrete demands associated with the management of perioperative irAEs, whose profile qualitatively differs from conventional surgical morbidity, and active participation in therapeutic decisions that are now built before and after the operating room, with tumor biology as the common language of the multidisciplinary team. Surgeons who incorporate these competencies not only operate in a more complex environment, but do so with better tools to offer each patient the best strategy for their tumor, their biology, and their clinical condition.

## Data Availability

The datasets generated and/or analyzed during the current study are available from the corresponding author upon reasonable request.

## References

[1] Abiko K, Matsumura N, Hamanishi J, Horikawa N, Murakami R, Yamaguchi K (2015). IFN-γ from lymphocytes induces PD-L1 expression and promotes progression of ovarian cancer. Br J Cancer.

[2] Al-Batran SE, Homann N, Pauligk C, Goetze TO, Meiler J, Kasper S (2019). Perioperative chemotherapy with fluorouracil plus leucovorin, oxaliplatin, and docetaxel versus fluorouracil or capecitabine plus cisplatin and epirubicin for locally advanced, resectable gastric or gastro-oesophageal junction adenocarcinoma (FLOT4): a randomised, phase 2/3 trial. Lancet.

[3] Ajani JA, D’Amico TA, Bentrem DJ, Chao J, Cooke D, Corvera C (2022). Gastric Cancer, Version 2.2022, NCCN Clinical Practice Guidelines in Oncology. J Natl Compr Canc Netw.

[4] André T, Tougeron D, Piessen G, de la Fouchardière C, Louvet C, Adenis A (2023). Neoadjuvant nivolumab plus ipilimumab and adjuvant nivolumab in localized deficient mismatch repair/microsatellite instability-high gastric or esophagogastric junction adenocarcinoma: the GERCOR NEONIPIGA phase II study. J Clin Oncol.

[5] Bang YJ, Kim YW, Yang HK, Chung HC, Park YK, Lee KH (2012). Adjuvant capecitabine and oxaliplatin for gastric cancer after D2 gastrectomy (CLASSIC): a phase 3 open-label, randomised controlled trial. Lancet.

[6] Bang YJ, Van Cutsem E, Feyereislova A, Chung HC, Shen L, Sawaki A (2010). Trastuzumab in combination with chemotherapy versus chemotherapy alone for treatment of HER2-positive advanced gastric or gastro-oesophageal junction cancer (ToGA): a phase 3, open-label, randomised controlled trial. Lancet.

[7] Boku N (2013). HER2-positive gastric cancer. Gastric Cancer.

[8] Bonenkamp JJ, Hermans J, Sasako M, van de Velde CJ, Welvaart K, Songun I (1999). Extended lymph-node dissection for gastric cancer. N Engl J Med.

[9] Brar G, Shah MA (2019). The role of pembrolizumab in the treatment of PD-L1 expressing gastric and gastroesophageal junction adenocarcinoma. Ther Adv Gastroenterol.

[10] Bray F, Laversanne M, Sung H, Ferlay J, Siegel RL, Soerjomataram I (2024). Global cancer statistics 2022: GLOBOCAN estimates of incidence and mortality worldwide for 36 cancers in 185 countries. CA Cancer J Clin.

[11] Cancer Genome Atlas Research Network (2014). Comprehensive molecular characterization of gastric adenocarcinoma. Nature.

[12] Chao J, Fuchs CS, Shitara K, Tabernero J, Muro K, Van Cutsem E (2021). Assessment of pembrolizumab therapy for the treatment of microsatellite instability-high gastric or gastroesophageal junction cancer among patients in the KEYNOTE-059, KEYNOTE-061, and KEYNOTE-062 clinical trials. JAMA Oncol.

[13] Choi YY, Kim H, Shin SJ, Kim HY, Lee J, Yang HK (2019). Microsatellite instability and programmed cell death-ligand 1 expression in stage II/III gastric cancer: post hoc analysis of the classic randomized controlled study. Ann Surg.

[14] Coburn N, Cosby R, Klein L, Knight G, Malthaner R, Mamazza J (2017). Staging and surgical approaches in gastric cancer: a clinical practice guideline. Curr Oncol.

[15] Cunningham D, Allum WH, Stenning SP, Thompson JN, Van de Velde CJ, Nicolson M (2006). Perioperative chemotherapy versus surgery alone for resectable gastroesophageal cancer. N Engl J Med.

[16] Cunningham D, Starling N, Rao S, Iveson T, Nicolson M, Coxon F (2008). Capecitabine and oxaliplatin for advanced esophagogastric cancer. N Engl J Med.

[17] D’Angelica M, Gonen M, Brennan MF, Turnbull AD, Bains M, Karpeh MS (2004). Patterns of initial recurrence in completely resected gastric adenocarcinoma. Ann Surg.

[18] Degiuli M, Reddavid R, Tomatis M, Ponti A, Morino M, Sasako M (2021). D2 dissection improves disease-specific survival in advanced gastric cancer patients: 15-year follow-up results of the Italian Gastric Cancer Study Group D1 versus D2 randomised controlled trial. Eur J Cancer.

[19] Dinis-Ribeiro M, Libânio D, Uchima H, Spaander MCW, Bornschein J, Matysiak-Budnik T (2025). Management of epithelial precancerous conditions and early neoplasia of the stomach (MAPS III): European Society of Gastrointestinal Endoscopy (ESGE), European Helicobacter and Microbiota Study Group (EHMSG) and European Society of Pathology (ESP) Guideline update 2025. Endoscopy.

[20] Fuchs CS, Doi T, Jang RW, Muro K, Satoh T, Machado M (2018). Safety and efficacy of pembrolizumab monotherapy in patients with previously treated advanced gastric and gastroesophageal junction cancer: phase 2 clinical KEYNOTE-059 trial. JAMA Oncol.

[21] Paoletti X, Oba K, Burzykowski T, Michiels S, Ohashi Y, GASTRIC (Global Advanced/Adjuvant Stomach Tumor Research International Collaboration) Group (2010). Benefit of adjuvant chemotherapy for resectable gastric cancer: a meta-analysis. JAMA.

[22] Hamashima C (2014). Current issues and future perspectives of gastric cancer screening. World J Gastroenterol.

[23] Hatta W, Ogata Y, Ogasawara K, Hatayama Y, Saito M, Kanno T (2025). Recent advances in endoscopic submucosal dissection for gastric cancer: focusing on expanded indications and technological innovations. J Gastric Cancer.

[24] Janjigian YY, Al-Batran SE, Wainberg ZA, Muro K, Molena D, Van Cutsem E (2025). Perioperative durvalumab in gastric and gastroesophageal junction cancer. N Engl J Med.

[25] Janjigian YY, Shitara K, Moehler M, Garrido M, Salman P, Shen L (2021). First-line nivolumab plus chemotherapy versus chemotherapy alone for advanced gastric, gastro-oesophageal junction, and oesophageal adenocarcinoma (CheckMate 649): a randomised, open-label, phase 3 trial. Lancet.

[26] Janjigian YY, Van Cutsem E, Muro K, Wainberg Z, Al-Batran SE, Hyung WJ (2022). MATTERHORN: phase III study of durvalumab plus FLOT chemotherapy in resectable gastric/gastroesophageal junction cancer. Future Oncol.

[27] Japanese Gastric Cancer Association (2026). Japanese gastric cancer treatment guidelines 2025 (7th edition). Gastric Cancer.

[28] Joshi DC, Sharma A, Prasad S, Singh K, Kumar M, Sherawat K (2024). Novel therapeutic agents in clinical trials: emerging approaches in cancer therapy. Discov Oncol.

[29] Koerner AS, Moy RH, Ryeom SW, Yoon SS (2023). The present and future of neoadjuvant and adjuvant therapy for locally advanced gastric cancer. Cancers (Basel).

[30] Kohlruss M, Chakraborty S, Hapfelmeier A, Jesinghaus M, Slotta-Huspenina J, Novotny A (2023). Low microsatellite instability: a distinct instability type in gastric cancer?. J Cancer Res Clin Oncol.

[31] Le DT, Durham JN, Smith KN, Wang H, Bartlett BR, Aulakh LK (2017). Mismatch repair deficiency predicts response of solid tumors to PD-1 blockade. Science.

[32] Lin Y, Liao Y, Shen J (2025). Addition of immunotherapy to perioperative chemotherapy for resectable gastric and gastroesophageal junction cancer: a meta-analysis of phase 2/3 trials. Front Immunol.

[33] Lordick F, Carneiro F, Cascinu S, Fleitas T, Haustermans K, Piessen G (2022). Gastric cancer: ESMO Clinical Practice Guideline for diagnosis, treatment and follow-up. Ann Oncol.

[34] Macdonald JS, Smalley SR, Benedetti J, Hundahl SA, Estes NC, Stemmermann GN (2001). Chemoradiotherapy after surgery compared with surgery alone for adenocarcinoma of the stomach or gastroesophageal junction. N Engl J Med.

[35] Marano L, Carbone L, Poto GE, Restaino V, Piccioni SA, Verre L (2023). Extended lymphadenectomy for gastric cancer in the neoadjuvant era: current status, clinical implications and contentious issues. Curr Oncol.

[36] Mocellin S, McCulloch P, Kazi H, Gama-Rodrigues JJ, Yuan Y, Nitti D (2015). Extent of lymph node dissection for adenocarcinoma of the stomach. Cochrane Database Syst Rev.

[37] Neumann PA, Berlet MW, Friess H (2021). Surgical oncology in the age of multimodality therapy for cancer of the upper and lower gastrointestinal tract. Expert Rev Anticancer Ther.

[38] Niimi T, Nagashima K, Ward JM, Minoo P, Zimonjic DB, Popescu NC (2001). claudin-18, a novel downstream target gene for the T/EBP/NKX2.1 homeodomain transcription factor, encodes lung- and stomach-specific isoforms through alternative splicing. Mol Cell Biol.

[39] Pardoll DM (2012). The blockade of immune checkpoints in cancer immunotherapy. Nat Rev Cancer.

[40] Peixoto RDA, Mathias-Machado MC, Jácome A, Gil M, Fogacci J, Sodré B (2023). PD-L1 testing in advanced gastric cancer-what physicians who treat this disease must know-a literature review. J Gastrointest Oncol.

[41] Pernomian LS, Teixeira MF, Araujo RLC, Uson PLS (2025). Perioperative immunotherapy in gastric cancer in the spotlight. World J Clin Oncol.

[42] Pietrantonio F, Miceli R, Raimondi A, Kim YW, Kang WK, Langley RE (2019). Individual patient data meta-analysis of the value of microsatellite instability as a biomarker in gastric cancer. J Clin Oncol.

[43] Raimondi A, Lonardi S, Murgioni S, Cardellino GG, Tamberi S, Strippoli A (2025). Tremelimumab and durvalumab as neoadjuvant or non-operative management strategy of patients with microsatellite instability-high resectable gastric or gastroesophageal junction adenocarcinoma: the INFINITY study by GONO. Ann Oncol.

[44] Rüschoff J, Hanna W, Bilous M, Hofmann M, Osamura RY, Penault-Llorca F (2012). HER2 testing in gastric cancer: a practical approach. Mod Pathol.

[45] Sahin U, Koslowski M, Dhaene K, Usener D, Brandenburg G, Seitz G (2008). Claudin-18 splice variant 2 is a pan-cancer target suitable for therapeutic antibody development. Clin Cancer Res.

[46] Sakuramoto S, Sasako M, Yamaguchi T, Kinoshita T, Fujii M, Nashimoto A (2007). Adjuvant chemotherapy for gastric cancer with S-1, an oral fluoropyrimidine. N Engl J Med.

[47] Sasako M, Sakuramoto S, Katai H, Kinoshita T, Furukawa H, Yamaguchi T (2011). Five-year outcomes of a randomized phase III trial comparing adjuvant chemotherapy with S-1 versus surgery alone in stage II or III gastric cancer. J Clin Oncol.

[48] Sasako M, Sano T, Yamamoto S, Kurokawa Y, Nashimoto A, Kurita A (2008). D2 lymphadenectomy alone or with para-aortic nodal dissection for gastric cancer. N Engl J Med.

[49] Schuhmacher C, Gretschel S, Lordick F, Reichardt P, Hohenberger W, Eisenberger CF (2010). Neoadjuvant chemotherapy compared with surgery alone for locally advanced cancer of the stomach and cardia: European Organisation for Research and Treatment of Cancer randomized trial 40954. J Clin Oncol.

[50] Shah MA, Shitara K, Ajani JA, Bang YJ, Enzinger P, Ilson D (2023). Zolbetuximab plus CAPOX in CLDN18.2-positive gastric or gastroesophageal junction adenocarcinoma: the randomized, phase 3 GLOW trial. Nat Med.

[51] Shitara K, Bang YJ, Wyrwicz LS, Rha SY, Oshima T, Pietrantonio F (2026). Surrogate endpoints for survival in KEYNOTE-585: neoadjuvant/adjuvant pembrolizumab plus chemotherapy versus placebo plus chemotherapy for gastric or gastroesophageal junction adenocarcinoma. ESMO Open.

[52] Shitara K, Lordick F, Bang YJ, Enzinger P, Ilson D, Shah MA (2023). Zolbetuximab plus mFOLFOX6 in patients with CLDN18.2-positive, HER2-negative, untreated, locally advanced unresectable or metastatic gastric or gastro-oesophageal junction adenocarcinoma (SPOTLIGHT): a multicentre, randomised, double-blind, phase 3 trial. Lancet.

[53] Shitara K, Rha SY, Wyrwicz L, Oshima T, Karaseva N, Osipov M (2025). Pembrolizumab plus chemotherapy versus chemotherapy as perioperative therapy in locally advanced gastric and gastroesophageal junction cancer: final analysis of the randomized, phase III KEYNOTE-585 study. J Clin Oncol.

[54] Singh P, Toom S, Huang Y (2017). Anti-claudin 18.2 antibody as new targeted therapy for advanced gastric cancer. J Hematol Oncol.

[55] Smalley SR, Benedetti JK, Haller DG, Hundahl SA, Estes NC, Ajani JA (2012). Updated analysis of SWOG-directed intergroup study 0116: a phase III trial of adjuvant radiochemotherapy versus observation after curative gastric cancer resection. J Clin Oncol.

[56] Smyth EC, Wotherspoon A, Peckitt C, Gonzalez D, Hulkki-Wilson S, Eltahir Z (2017). Mismatch repair deficiency, microsatellite instability, and survival: an exploratory analysis of the Medical Research Council Adjuvant Gastric Infusional Chemotherapy (MAGIC) trial. JAMA Oncol.

[57] Topalian SL, Taube JM, Pardoll DM (2020). Neoadjuvant checkpoint blockade for cancer immunotherapy. Science.

[58] Wang FH, Zhang XT, Li YF, Tang L, Qu XJ, Ying JE (2021). The Chinese Society of Clinical Oncology (CSCO): Clinical guidelines for the diagnosis and treatment of gastric cancer, 2021. Cancer Commun (Lond).

[59] Ychou M, Boige V, Pignon JP, Conroy T, Bouché O, Lebreton G (2011). Perioperative chemotherapy compared with surgery alone for resectable gastroesophageal adenocarcinoma: an FNCLCC and FFCD multicenter phase III trial. J Clin Oncol.

[60] Yuan H, Bao M, Chen M, Fu J, Yu S (2025). Advances in immunotherapy and targeted therapy for gastric cancer: a comprehensive review. Br J Hosp Med (Lond).

[61] Zhao M, Yan CY, Wei YN, Zhao XH (2023). Breaking the mold: overcoming resistance to immune checkpoint inhibitors. Antiviral Res.

